# Exercise training using hybrid assistive limb (HAL) lumbar type for locomotive syndrome: a pilot study

**DOI:** 10.1186/s12891-021-04421-3

**Published:** 2021-06-12

**Authors:** Kousei Miura, Masao Koda, Kazuhiro Tamaki, Masatoshi Ishida, Aiki Marushima, Toru Funayama, Hiroshi Takahashi, Hiroshi Noguchi, Kentaro Mataki, Yoshihiro Yasunaga, Hiroaki Kawamoto, Yoshiyuki Sankai, Akira Matsumura, Masashi Yamazaki

**Affiliations:** 1 Department of Orthopaedic Surgery, Faculty of Medicine, University of Tsukuba, 1-1-1 Tennodai, Tsukuba, Ibaraki, 305-8575 Japan; 2 Medical Corporation, Kanjinkai, 3-35-13, Kamidaira, Fussa, Tokyo, 197-0012 Japan; 3 Eijyu Care Center, 1-2-30, Uriwariminami, Hirano, Osaka, 547-0023 Japan; 4 Department of Neurosurgery, Faculty of Medicine, University of Tsukuba, 1-1-1 Tennodai, Tsukuba, Ibaraki, 305-8575 Japan; 5 Center for Cybernics Research, University of Tsukuba, 1-1-1 Tennodai, Tsukuba, Ibaraki, 305-8575 Japan; 6 Ibaraki Prefectural University of Health Sciences, 4773 Ami, Inashiki-gun, Ibaraki, 300-0331 Japan

**Keywords:** Locomotive syndrome, Hybrid assistive limb, Lumbar type, Low back pain, Elderly people

## Abstract

**Background:**

With a rapidly aging population in Japan, locomotive syndrome is becoming an increasingly serious social problem. Exercise therapy using the lumbar type HAL, which is a wearable robot suit that can assist voluntary hip joint motion, would be expected to cause some beneficial effects for people with locomotive syndrome. The purpose of this study was to assess whether the deterioration of low back pain and any other adverse events would occur following HAL exercise therapy. Moreover, the changes of motor ability variables were evaluated.

**Methods:**

We enrolled 33 participants (16 men, 17 women) with locomotive syndrome in this study. They received exercise training (sit-to-stand, lumbar flexion-extension, and gait training) with HAL (in total 12 sessions). We assessed the change of low back pain (lumbar VAS). More than 50% and 25 mm increase compared to baseline was defined as adverse events. One-leg standing time (OLST), 10-m walking test (10MWT), Timed Up and Go test (TUG), 1-min sit-to-stand test (1MSTS), FIM mobility scores and EQ-5D were measured.

**Results:**

Of the 33 participants, 32 (16 men, 16 women) (97.0%) completed all 12 exercise training sessions using the lumbar type HAL. One woman aged 82 years withdrew because of right upper limb pain after the second session regardless of the use of HAL. There was no participant who had deterioration of low back pain. Any other adverse events including external injuries and/or falling, skin disorders, uncontrollable cardiovascular or respiratory disorders, and other health disorders directly related to this exercise therapy did not occur. Several outcome measures of motion ability including OLST, TUG and 1MSTS, EQ VAS and lumbar pain improved significantly after this HAL training.

**Conclusions:**

Almost all patients with locomotive syndrome completed this exercise training protocol without any adverse events related to HAL. Furthermore, balance function variables including OLST, TUG and 1MSTS improved after this HAL exercise therapy even though mobility function variables including 10MWT and FIM mobility scores did not show any significant change. These findings suggest that the exercise therapy using the lumbar type HAL would be one of the options for the intervention in locomotive syndrome.

## Background

There are 34 million elderly people in Japan representing 27% of the population [1] . With a rapidly aging population in Japan, elderly patients with disorders relating to motor function are becoming an increasingly serious social problem [2]﻿. The Japanese Orthopaedic Association (JOA) proposed the term “locomotive syndrome” in 2007. Locomotive syndrome is defined as a condition in which people have loss of mobility caused by degeneration of locomotive organs and require nursing care services due to disorders related to motor function [3, 4]. In Japanese older than 80 years, the prevalence of locomotive syndrome has reached 60% in men and 75% in women [5]. An aging society will be an important issue in the future, not only in Japan, but worldwide [6]. To improve loss of mobility, various exercise therapies have been conventionally performed. More importantly, less invasive therapies such as exercise with the use of robotic-assisted devices are required for elderly people to prevent injury due to excessive physical load.

**Fig. 1:**
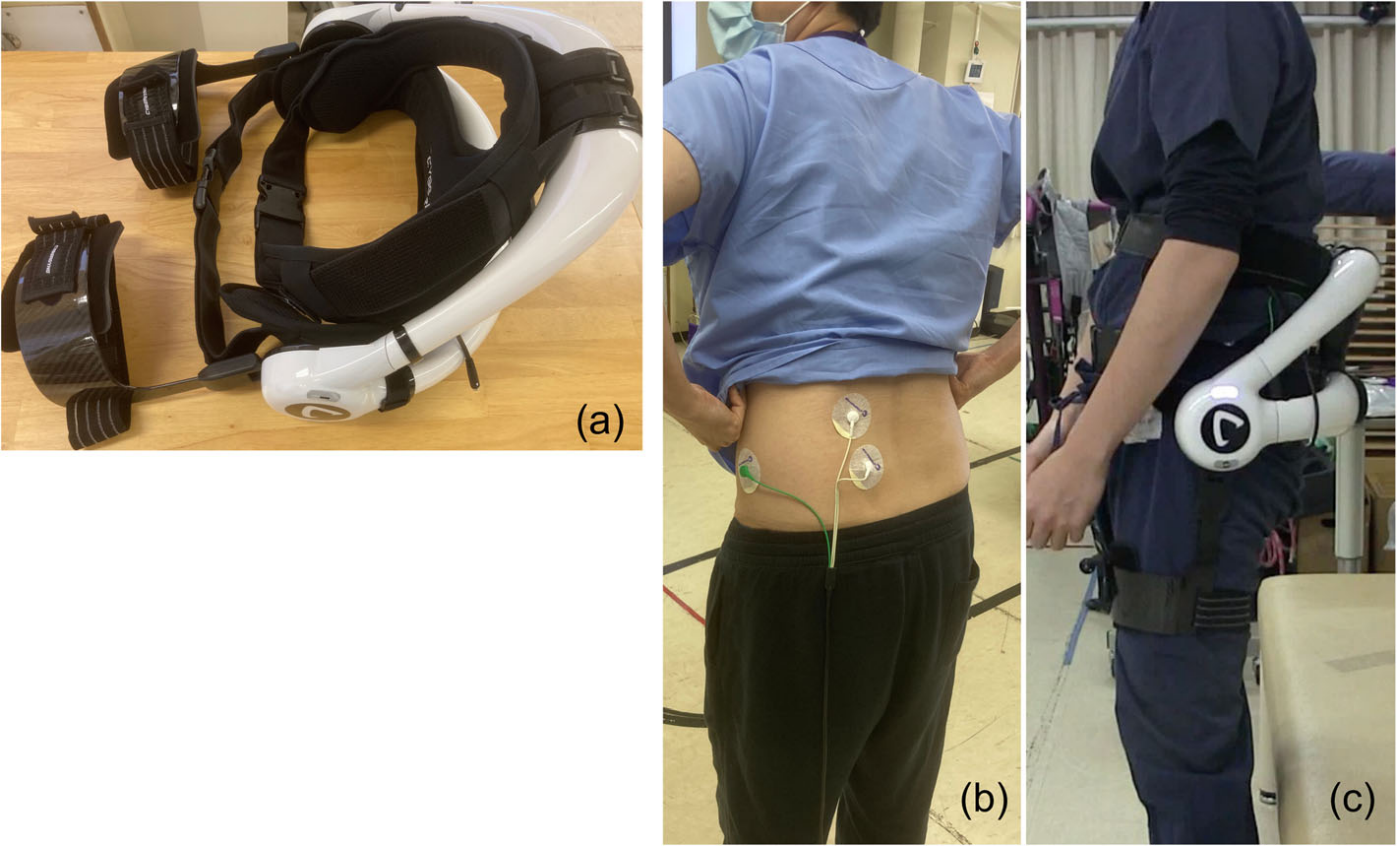
**a** Overview of the lumbar type hybrid assistive limb (HAL). **b** Photograph illustrating electrodes on the skin over lumbar erector spinae muscle to detect muscle action potential. **c** Lateral image of the worn lumbar type HAL

As for robotic-assisted device for rehabilitation, robot-assisted gait training has been recently drawing increasing attention. The Lokomat (Hocoma AG, Volketswil, Switzerland) has been used for the treatment of patients with spinal cord injury and stroke [7]. Some reviews showed the effectiveness of the Lokomat for these diseases [8, 9]. Although the Lokomat can assist walking movements with the support of body weight, it has the several disadvantages. The Lokomat is not able to assist any movements other than walking and is not portable because of its huge size. Thus, we focused our attention on the exercise therapy by using the lumbar type hybrid assistive limb (HAL) (Cyberdyne Inc., Ibaraki, Japan) (Fig. 1). HAL is the wearable robot suit that can assist joint motion. It can provide voluntary joint motion assist with the reaction to the wearer’s intention of standing up by detecting nerve and muscle action potentials of the lumbar erector spinae muscles through electrodes attached on the skin. To date, several types of HAL (HAL for lower limbs and HAL for single joints) have been applied for several musculoskeletal and neurological disorders [10–14]. It has been reported that the lower limb type HAL, which can assist hip and knee joint motion, might improve gait function for myelopathy [10], cerebral infarction [11], and cerebral palsy [12]. The single-joint type HAL can assist knee and elbow joint motion. Several clinical case reports of exercise therapy using the single-joint type HAL for cerebral palsy and brachial plexus injury have been reported [13, 14].

To date, it has been shown the possibility of reduction of the subjective lumbar fatigue and the improvement in lifting performance during repetitive lifting movements [15], repetitive snow-shoveling movements [16], and simulated patient transfer movements [17] in healthy adults with the lumbar type HAL. Moreover, cardiopulmonary burden during stand-up exercise may be reduced because of the use of the lumbar type HAL among healthy volunteers [18]. In addition, the lumbar type HAL is portable and able to assist voluntary joint motion during not only walking but also sit-to-stand and lumbar flexion-extension unlike the Lokomat.

We hypothesized that exercise therapy using the lumbar type HAL would cause some effects on motor function for people with locomotive disorders. However, there are few reports of the use of the lumbar type HAL for those people. The purpose of this pilot study was to assess the low back pain and adverse events following exercise therapy with the lumbar type HAL for safety evaluation. Moreover, we evaluated several outcome measures regarding motor ability and QOL to design advanced clinical trials for ensuring the effects of lumbar type HAL on locomotive syndrome in the future.

## Methods

### Participants

This was a retrospective, single-arm, exploratory study conducted at our institutes between April 2018 and March 2019. The inclusion criteria were as follows: (1) admitted to nursing facilities or the use of day care; (2) aged 40 years or more; (3) at least one affirmative answer on loco-check; (4) suitable body size to fit the lumbar type HAL (maximum waist circumference, 120 cm; maximum thigh circumference, 80 cm). Loco-check is a self-assessment questionnaire composed of the following 7 items to evaluate the locomotive syndrome: (1) You cannot put on a pair of socks while standing on one leg; (2) You stumble or slip in your house; (3) You need to use a handrail when going upstairs; (4) You cannot get across the road at a crossing before the traffic light changes; (5) You have difficulty walking continuously for 15 min; (6) You find it difficult to walk home carrying a shopping bag weighing about 2 kg (e.g., two 1-l milk packs); (7) You find it difficult to do housework requiring physical strength (e.g., use of vacuum cleaner to clean the rooms, putting futons into and taking them out of the closet, etc.) [4]. The exclusion criteria were as follows: (1) skin disorders prohibiting the attachment of the electrodes; (2) inadequately controlled cardiovascular or respiratory disorders interfering with exercise therapy; (3) severe dementia that prohibits understanding of the training program using HAL; (4) lower limb joints disorders that may seriously affect the results of this study.

We obtained informed consent from all participants. This study was approved by the institutional Review Board of our institution (IRB approval No. H29–253) and was performed in accordance with the Declaration of Helsinki.

### The lumbar type HAL

The lumbar type HAL consists of an exoskeletal frame, power units, and lumbar and thigh molds. Actuators of power units, which are located on the wearer’s bilateral femoral greater trochanters, generate a torque assisting hip extension motion. A triaxial accelerometer within the exoskeletal frame can detect the absolute trunk angle and angular sensors within the power units and potentiometers can detect the relative angles of the hip joints. Through these mechanisms, the lumbar type HAL is able to support the wearer’s motions with coordination of the level and timing of the torque. Additionally, the lumbar type HAL has two hybrid control systems as follows: a cybernic voluntary control (CVC) and a cybernic autonomous system (CAC). Hara et al. [19] reported these two control systems in detail. CVC system can control the actuator torque of the HAL to augment joint torque of the wearer according to voluntary muscle activity by detecting wearer’s motion through myoelectricity. On the other hand, CAC system can support the wearer’s weight for reducing moment caused by trunk flexion as a gravity compensation algorithm.

Because of simpler construction compared to the lower limb type HAL, the lumbar type HAL has the advantage of lightness, weighing only 2.9 kg including the battery. Besides, wearers can equip the lumbar type HAL by themselves in about 5 min.

### Training program

**Fig. 2:**
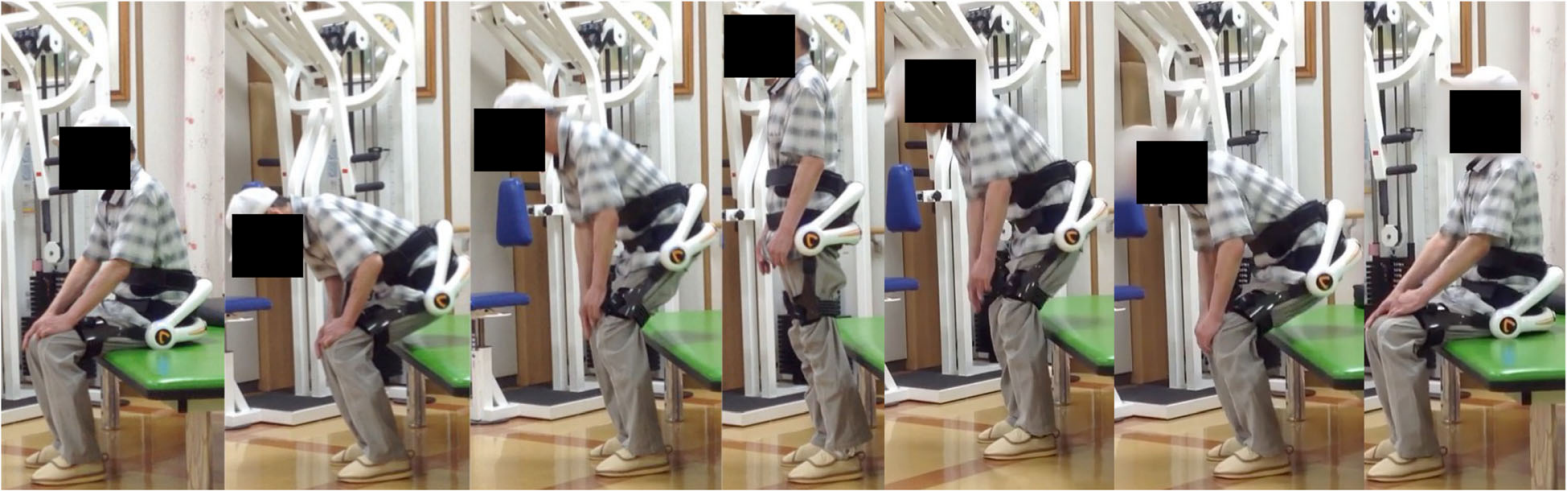
Repetitive sit-to-stand training with the lumbar type HAL

**Fig. 3:**
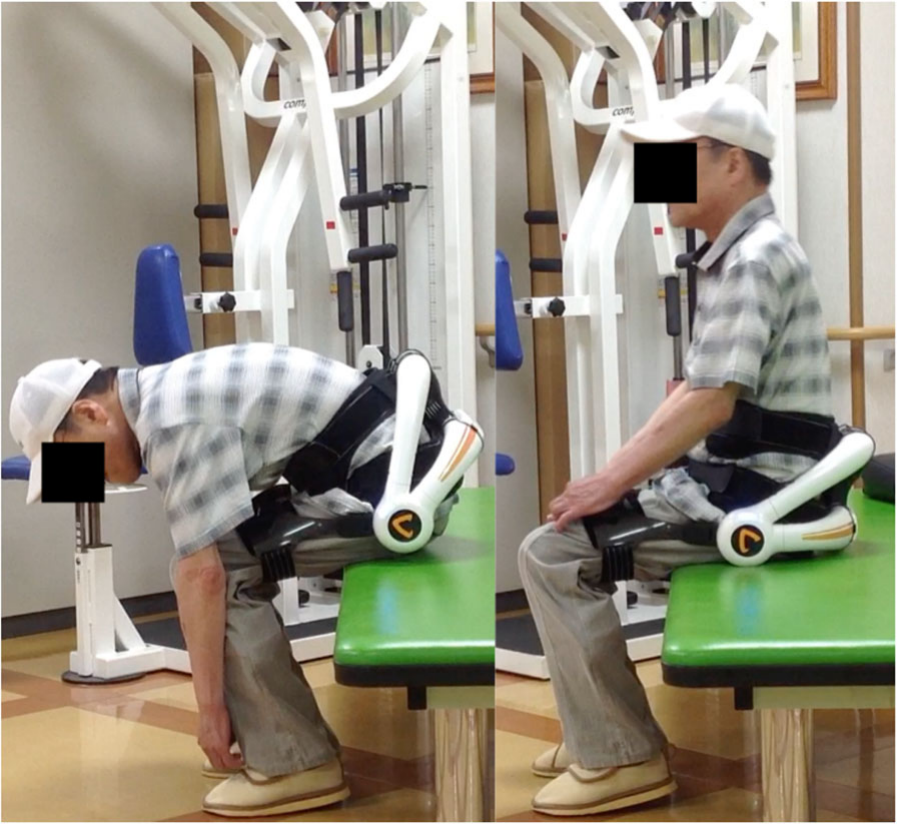
Repetitive lumbar flexion and extension training with the lumbar type HAL

**Fig. 4:**
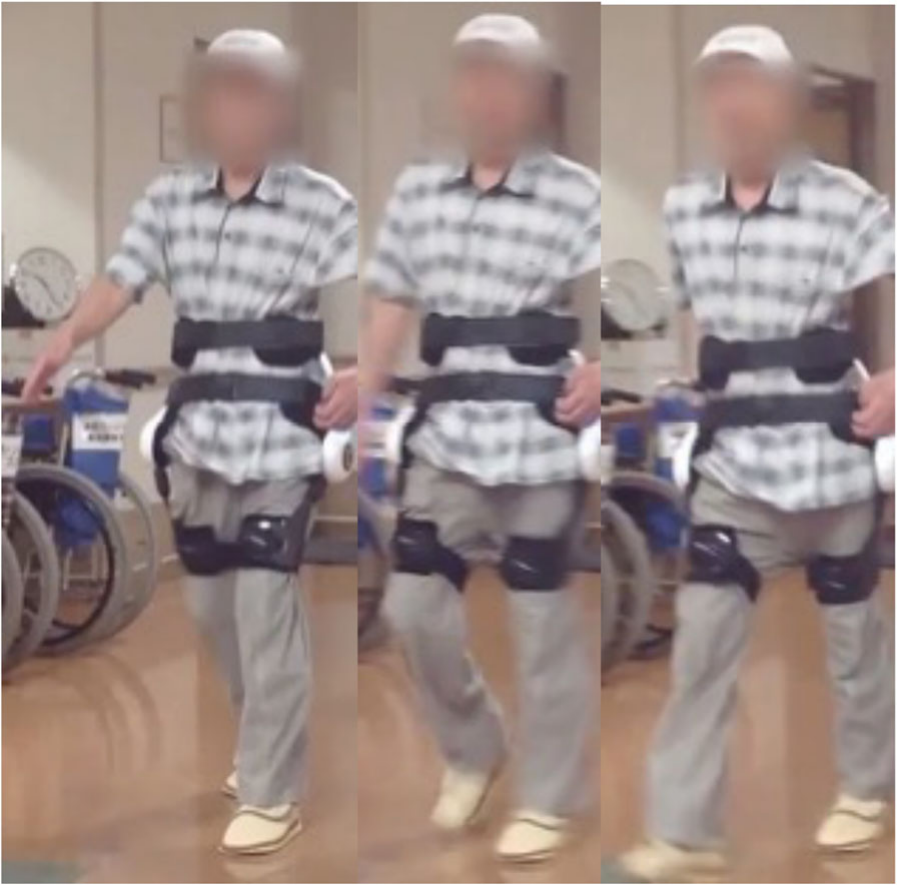
Continuous gait training on the ground with the lumbar type HA

Exercise training with the lumbar type HAL comprised sit-to-stand training, lumbar flexion–extension training, and gait training. Each about 5 min were needed for fitting and removal of the lumbar type HAL. Repetitive sit-to-stand training with the lumbar type HAL was performed for 10 min (Fig. 2). Participants were allowed to push themselves with their hands to stand up if they had difficulty standing up without any assistance. After that, repetitive lumbar flexion and extension motion was performed for 5 min in a sitting position (Fig. 3). Finally, gait training on the ground was performed continuously using the lumbar type HAL at a pace comfortable for the participant for 5 min (Fig. 4). If necessary, participants were allowed to use a cane or a walker during gait training. A rest time of 5 min was set between each training. Each training combined sit-to-stand, lumbar flexion–extension and gait training 3 times per week for 4 weeks (a total of 12 sessions).

### Outcome measures

A retrospective analysis of prospectively collected data was performed. We assessed the change of low back pain by using visual analogue scale (VAS) (lumbar VAS). More than 50% and 25 mm increase compared to baseline was defined as adverse events. Moreover, we also defined newly development of the following health disorders related to exercise with the lumbar type HAL as adverse events: (1) external injuries and/or falling during exercise therapy; (2) skin disorders related to wearing HAL; (3) uncontrollable cardiovascular or respiratory disorders interfering with exercise therapy; (4) any other health disorder directly related to this exercise therapy.

Motor ability and quality of life (QOL) were also evaluated at baseline within 1 week before HAL training (pre-HAL) and post-intervention within 1 week after HAL training (post-HAL). Motor ability was assessed with a one-leg standing time (OLST), a 10-m walking test (10MWT), a Timed Up and Go test (TUG), 1-min sit-to-stand test (1MSTS), mobility scores (locomotion and stair climb) from the Functional Independence Measure (FIM) assessment tool, and the number of affirmative answers on loco-check. As for OLST, we calculated the average of both sides of lower limb. OLST, TUG and 1MSTS was evaluated only one time without the lumbar type HAL. QOL was assessed using the EuroQOL-5d (EQ-5D) and EQ-Visual Analog Scale (EQ-VAS) [20]. Besides, limb pain was evaluated as the pain in locomotive organ (limb VAS).

### Statistical analysis

Some missing data were replaced by baseline observations carried forward. A repeated measures ANOVA was used to evaluate the differences between pre- and post-HAL exercise training. All statistical analyses were conducted using the JMP software package ver. 14.0.0 (SAS Institute., Cary, NC, USA), and for all comparisons *P* < 0.05 was considered a significant difference.

## Results

We prospectively enrolled 33 participants (16 men, 17 women) with locomotive syndrome in the present study. Their mean age ± SD was 77 ± 10 years (range, 40–95 y), their mean height ± SD was 155 ± 7.6 cm (140–178 cm), and their weight ± SD was 55 ± 9.6 kg (39–78 kg). The medical comorbidities affecting locomotive function were lumbar spondylosis in 13 cases, cerebrovascular disease in 12 cases, lumbar canal stenosis in 8 case, osteoarthritis of the knee in 7 cases, osteoporosis in 7 cases, osteoporotic vertebral fracture in 5 cases, parkinsonism in 3 cases, proximal femoral fracture in 2 cases, cervical spondylotic myelopathy in 1 case, and spinal cord injury in 1 case.

Of the 33 participants, 32 (16 men, 16 women) (97.0%) completed all 12 exercise training sessions using the lumbar type HAL. One woman aged 82 years withdrew because of right upper limb pain after the second session. Her exercise therapy was interrupted because she hoped to quit it. Her right upper limb pain was diagnosed as muscular pain and it was spontaneously relieved without any intervention. She needed a walker to walk. Excessive load by walking exercise regardless of the use of HAL on her upper limb to bring the walker might cause her upper limb pain. Of 32 participants that completed the exercise training with HAL, there was no participant who had deterioration of low back pain. Any other adverse events including external injuries and/or falling, skin disorders, uncontrollable cardiovascular or respiratory disorders, and other health disorders directly related to this exercise therapy did not occur.

The comparison of outcome measures is summarized in Table 1. Regarding motor ability, OLST significantly increased after HAL training and exhibited medium effect size (d = 0.76). TUG time significantly decreased after HAL training and showed very large effect size (d = 0.96). 1MSTS exhibited significant increase and very large effect size (d = 0.99). The number of affirmative answers on loco-check significantly decreased after HAL training and showed medium effect size. The answers of participants to loco-check are summarized in Table 2. As a result, 13 participants (40.6%) changed their answers from ‘Yes’ to ‘No’ on one or more loco-check questionnaire items. On the other hand, 10MWT and in FIM mobility score did not differ significantly. As for QOL, EQ-VAS significantly increased after HAL training and showed medium effect size (d = 0.53). However, EQ-5D did not present significant change. Lumbar VAS significantly improved after HAL training and exhibited large effect size (d = 0.83), while limb VAS did not change significantly.

**Table 1:** Comparison of outcome measures between pre- and post-HAL exercise training (*n* = 32)

		Pre-HAL		Post-HAL		
Outcome measures		Mean ± SD	95% CI	Mean ± SD	95% CI	Effect size
Motor ability	OLST (s)	3.8 ± 6.7	1.13–6.39	6.9 ± 11.5*	2.31–11.4	0.76
	10MWT (s)	15.2 ± 8.0	11.9–18.5	14.9 ± 9.1	11.1–18.6	0.06
	TUG (s)	20.7 ± 9.3	17.2–24.2	17.7 ± 7.2^†^	15.1–20.4	0.96
	1MSTS (times)	15.1 ± 6.5	12.7–17.6	18.0 ± 6.0^†^	15.7–20.2	0.99
	FIM mobility scores	11.0 ± 2.6	10.1–11.9	11.5 ± 2.6	10.5–12.4	0.05
	The number of affirmative answers on loco-check	4.6 ± 1.7	4.0–5.2	4.2 ± 1.7*	3.6–4.7	0.71
QOL	EQ5D	0.78 ± 0.11	0.73–0.82	0.80 ± 0.12	0.76–0.85	0.41
	EQ VAS	46.4 ± 18.4	39.8–53.0	59.3 ± 22.2*	51.9–67.9	0.53
Pain in locomotive Organs	Lumbar VAS	33.1 ± 31.5	21.8–44.2	24.9 ± 28.6*	14.8–35.1	0.83
	Limb VAS	32.4 ± 26.3	23.1–41.8	26.6 ± 22.5	18.6–34.6	0.46

*OLST* one-leg standing time, *10MWT* 10-m walking test, *TUG* timed up and go test, *1MSTS* 1-min sit-to-stand test, *EQ5D* EuroQOL-5d

*P < 0.05, †*P* < 0.01

**Table 2:** Patricipants’ answers to Loco-check between pre- and post-HAL exercise training

Loco-check	Pre-HAL		Post-HAL	
	Yes	No	Yes	No
(1) You cannnot put on a pair of socks while standing on one leg	30	2	30	2
(2) You stumble or slip in your house	12	20	7	25
(3) You need to use a handrail when going upstairs	31	1	30	2
(4) You cannot get across the road at a crossing before the traffic light changes	22	10	21	11
(5) You have difficulty walking continuously for 15 min	22	10	20	12
(6) You find it difficult to walk home carrying a shopping bag weighing about 2 kg	18	14	13	19
(7) You find it difficult to do housework requiring physical strength	11	21	9	23

## Discussion

This study showed that 32 of 33 (97.0%) participants with locomotive syndrome completed exercise therapy with the use of the lumbar type HAL except for one 82-year-old woman who hoped to quit it due to upper limb pain after the second session regardless of the use of the HAL. The deterioration of low back pain and any other adverse events did not happen in all participants who completed this exercise therapy. However, there are several patients with locomotive syndrome needed help to equip the lumbar type HAL despite that healthy people can equip by themselves. It would be preferable that it should become easy-to-wear for locomotive syndrome. In addition, several motor function variables including OLST, TUG, 1MSTS improved after HAL training. In particular, 1MSTS and TUG time showed very large effect size. On the other hand, no significant difference was observed in 10MWT and FIM mobility scores. FIM mobility scores are composed of 50-m walking ability and stair climb ability. Thus, FIM mobility scores and 10MWT have been recognized as the evaluation tools of mobility function [21, 22]. On the other hand, OLST, TUG, 1MSTS have been recognized as the evaluation tools of balance function [23, 24]. Therefore, it is suspected that this exercise therapy using the lumbar type HAL may be more helpful for improving the balance function than for improving the mobility function in locomotive syndrome. Regarding lower limb type HAL, it has been reported that walking balance improved after gait training with it [25]. Similarly, repetitive sit-to-stand training and repetitive lumbar flexion and extension training with the use of lumbar type HAL are believed to improve balance function. The lumbar type HAL can provide voluntary hip joint motion assist with the reaction to the wearer’s intention of standing up. The characteristic may cause the effect on balance function. However, the detailed mechanisms of that improvement are unclear. Further work such as kinematics analysis is needed to explore the mechanism.

Regarding conventional exercise therapy for locomotive syndrome, other studies have investigated the effects of one-leg stands training and squats training on the elderly. These are considered to be safe and feasible exercises at home [26].

Aoki et al. reported that 87 of 97 elderly participants (89.7%) completed the exercise training (one-leg stands and squats training) performed for 3 months, and physical function such as OLST and 5 times sit-to-stand tests and 7 of 8 scores of the SF-8 improved significantly [27]. Ishibashi et al. reported that 97 of 151 elderly women (64.2%) completed one-leg stands and squats training for 2 months, and physical function including OLST, 10 m maximal gait speed, and knee extension torque improved significantly [28]. Hashimoto et al. reported that 55 of 60 elderly people (91.7%) completed one-leg stands and squats training for 3 months, and OLST improved significantly [29]. Regarding exercise therapy for locomotive syndrome, it has been reported that gradual increase of the exercise load for people with chronic locomotive degeneration is important for safety [30]. Nevertheless, a high load is required for any functional improvement [26]. Thus, delicate adjustments of exercise load should be considered while exercise training for locomotive syndrome.

Recent advance in robotic-assisted device for rehabilitation including HAL has been remarked. To our best knowledge, a few clinical studies have reported on the robotic-assisted exercise therapy for locomotive syndrome. Kotani et al. [31] firstly reported core exercise and squat exercise using the lumbar type HAL showed significant improvement of motor function in 16 frailty patients including 8 Parkinson’s disease (PD) patients and 8 non-PD patients with spine problems. In this study, three kinds of training including sit-to-stand training, lumbar flexion–extension training, and gait training were performed by using the lumbar type HAL for 33 locomotive syndrome patients. Additionally, QOL was assessed in addition to motor function and pain. The completion rate of the exercise therapy with the use of lumbar type HAL was relatively higher compared to previous reported conventional exercise therapy. All participants who completed the HAL exercise training did not develop any adverse events including the increase of low back pain. Moreover, balance function variables including OLST, TUG and 1MSTS improved after HAL exercise therapy similar to previous studies about conventional exercise therapy. As for pain, low back pain and limb pain were evaluated separately. Only low back pain significantly decreased after HAL training. EQ-VAS also showed significant improvement, which might reflect some positive effects on QOL in participants with locomotive syndrome. Presumably, these results are partly due to the special characteristic of HAL to be able to support the wearer’s motions with coordination of the level and timing of the torque by detecting nerve and muscle action potentials of the lumbar elector spinae muscles. The coordinated voluntary joint motion assist with the wearer’s intention by the lumbar type HAL is considered to induce positive feedback to the wearer’s nerve and muscle. We speculate that this novel mechanism of the lumbar type HAL can provide optimal exercise load for locomotive syndrome with loss of mobility. Our results suggest that this exercise training using the lumbar type HAL is promising for locomotive syndrome.

There are several limitations to the present study. First, we did not include a control group. The possibility cannot be excluded that only higher-intensity exercise could improve even outcome measures requiring higher motor function regardless of the lumbar type HAL. Next, we could not obtain kinematical measures such as motion analysis and electromyographical measures to assess whether the HAL could suitable exercise load to improve motor ability. Thus, the effect suggested by this HAL exercise training for locomotive syndrome should not be generalized. Furthermore, we had no available follow up data. These data are important for evaluating the long-term outcome of this protocol of exercise training using the lumbar type HAL. Further studies on these issues are necessary to clarify the effect of the lumbar type HAL for locomotive syndrome.

## Conclusion

Our results revealed that 32 of 33 (97.0%) participants with locomotive syndrome completed exercise therapy with the use of the lumbar type HAL. The deterioration of low back pain and any other adverse events did not occur in all participants who completed this exercise therapy. Regarding motor ability, balance function variables including OLST, TUG and 1MSTS improved after this HAL exercise therapy even though mobility function variables including 10MWT and FIM mobility scores did not show any significant change. Furthermore, lumbar VAS and EQ-5D significantly improved after exercise therapy with HAL. These findings suggest that the exercise therapy using the lumbar type HAL would be one of the options for the intervention in locomotive syndrome. Further studies are needed to elucidate the effect of the exercise therapy using the lumbar type HAL for locomotive syndrome.

## Data Availability

The datasets generated and/or analysed during the current study are not publicly available due to limitations of ethical approval involving the patient data and anonymity but are available from the corresponding author on reasonable request.

## Abbreviations

HAL: Hybrid assistive limb
OLST: One-leg standing time
10MWT: 10-m walking test
TUG: Timed up and go test
1MSTS: 1-min sit-to-stand test
JOA: Japanese Orthopaedic Association
CVC: Cybernic voluntary control
CAC: Cybernic autonomous system
QOL: Quality of life
FIM: Functional independence measure
EQ-5D: EuroQOL-5d
EQ-VAS: EQ-visual analog scale
